# Supplementary material for: No evidence for neonicotinoid preferences in the bumblebee Bombus impatiens
Source: R Soc Open Sci. 2020 May 20;7(5):191883. doi: 10.1098/rsos.191883 (PMC7277277; doi:10.1098/rsos.191883)
Supplement: Supplementary Material [file RSOS191883supp1.docx]

**Supplementary Material**

**Experiment 1,** *Controls for Evaporation*

In order to control for different rates of evaporation, we carried out a number of controls (alongside experimental bees) where we set up preference tubes in the same way as for the experiment, but without a bee present (sample sizes shown in Table S1). We measured the evaporation in these control tubes at the same time points as the experimental tubes.

To determine if evaporation varied by solution type, we carried out a GLMM with a Poisson distribution, with the response variable “amount consumed” and the explanatory variables “sucrose concentration” (ordinal factor, 4 levels), “imidacloprid concentration” (ordinal factor, 3 levels), “solution type” (neonicotinoid or control) and the random factor “preference tube” (because as with experimental bees, the 2 feeding tubes were paired within a larger preference tube). Because evaporation was minimal at earlier time points, we analysed evaporative control data for 24 hours only.

We found that solutions evaporated more depending on sucrose concentration (i.e. water evaporating the fastest, and 30% sucrose the slowest; *χ*^2^_3_ = 95.53; *p* < 0.0001; Fig. S1). Whether the solution contained neonicotinoid (*χ*^2^_1_ = 0.13; *p* = 0.72) and the neonicotinoid (or acetone) concentration (*χ*^2^_2_ = 4.92; *p* = 0.09) did not affect evaporation.

We then pooled the evaporative control data within each sucrose concentration treatment, taking an average for each time point (to subtract from experimental treatments); by having the larger sample sizes (sucrose concentration (%) n: 0=53; 5=69; 15=62; 30=66), it meant that we could ensure that the rates of evaporation were as accurate and precise as possible (i.e. if we had taken averages within each of the 24 solutions, some of these may have been biased by chance alone, which would in turn have biased all experimental solutions when we subtracted these values).

**Table S1**: Sample sizes for evaporative control solutions used in Experiment 1.

| **Sucrose concentration (%)** | **Imidacloprid/acetone control concentration (µg/kg)** | **Solution type** | **n** |
| --- | --- | --- | --- |
| 0 | 0.25 | neonic | 10 |
| 0 | 0.25 | sucrose | 12 |
| 0 | 1 | neonic | 7 |
| 0 | 1 | sucrose | 8 |
| 0 | 10 | neonic | 8 |
| 0 | 10 | sucrose | 8 |
| 5 | 0.25 | neonic | 12 |
| 5 | 0.25 | sucrose | 11 |
| 5 | 1 | neonic | 11 |
| 5 | 1 | sucrose | 12 |
| 5 | 10 | neonic | 12 |
| 5 | 10 | sucrose | 11 |
| 15 | 0.25 | neonic | 13 |
| 15 | 0.25 | sucrose | 12 |
| 15 | 1 | neonic | 8 |
| 15 | 1 | sucrose | 7 |
| 15 | 10 | neonic | 10 |
| 15 | 10 | sucrose | 12 |
| 30 | 0.25 | neonic | 12 |
| 30 | 0.25 | sucrose | 11 |
| 30 | 1 | neonic | 12 |
| 30 | 1 | sucrose | 11 |
| 30 | 10 | neonic | 12 |
| 30 | 10 | sucrose | 8 |

**Experiment 1: Supplemental methods and results**

**Table S2**: Number of bees per treatment that were excluded for not consuming a sufficient amount of solution to be included (details in main manuscript text).

|  |  | Imidacloprid concentration (µg/ kg) | | |
| --- | --- | --- | --- | --- |
|  |  | **0.25** | **1** | **10** |
| Sucrose concentration (%) | **0** | 20 | 10 | 15 |
|  | **5** | 2 | 6 | 6 |
|  | **15** | 1 | 0 | 1 |
|  | **30** | 0 | 2 | 0 |

**Table S3**: Concentrations used for each treatment in PPB (µg/ litre).

|  |  | Imidacloprid concentration (µg/ kg) | | |
| --- | --- | --- | --- | --- |
|  |  | **0.25** | **1** | **10** |
| Sucrose concentration (%) | **0** | 0.25 | 1.00 | 10.00 |
|  | **5** | 0.25 | 1.02 | 10.18 |
|  | **15** | 0.26 | 1.06 | 10.58 |
|  | **30** | 0.28 | 1.12 | 11.25 |


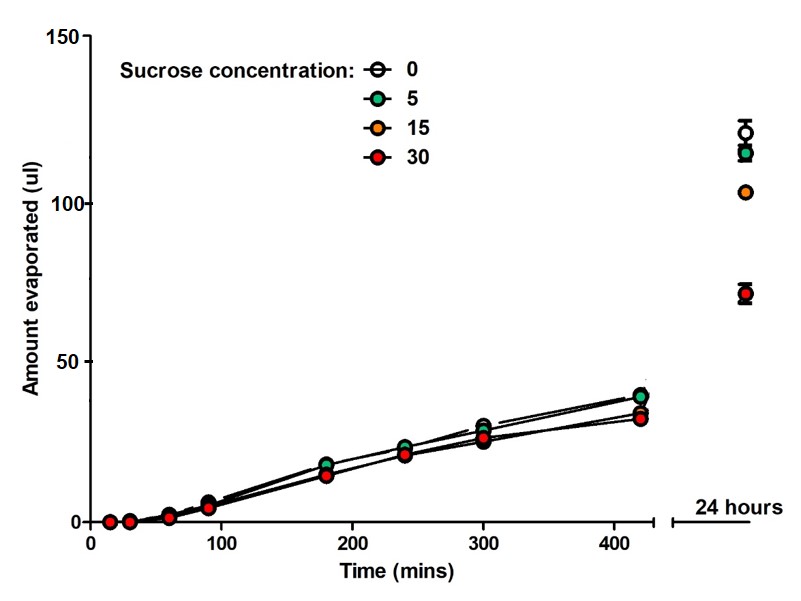


**Figure S1**: The rate of evaporation in control solutions across time. Data shown are mean values ±SEM within each sucrose concentration (the only variable that affected evaporation); error bars are not shown in many cases because they are too small.


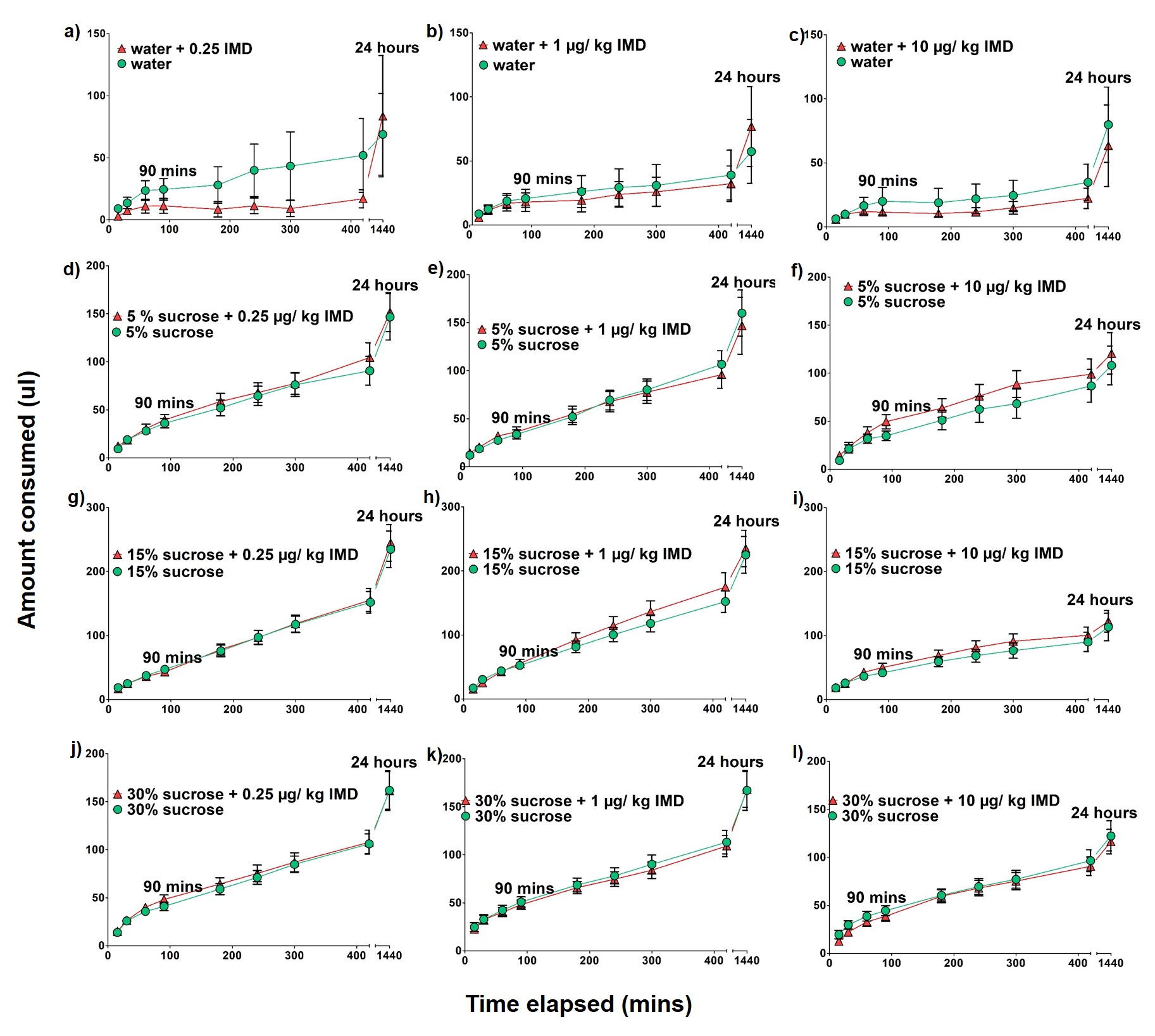


**Figure S2**: The cumulative amount (means ±SEM) that bees consumed of the neonicotinoid-containing solution versus the control solution, for all neonicotinoid and sucrose concentrations (12 treatments total) in Exp. 1. Note that axes differ between, but not within, each sucrose concentration treatment. Left to right across rows show treatments increasing in imidacloprid concentration. Top to bottom in columns show treatments increasing in sucrose concentration.


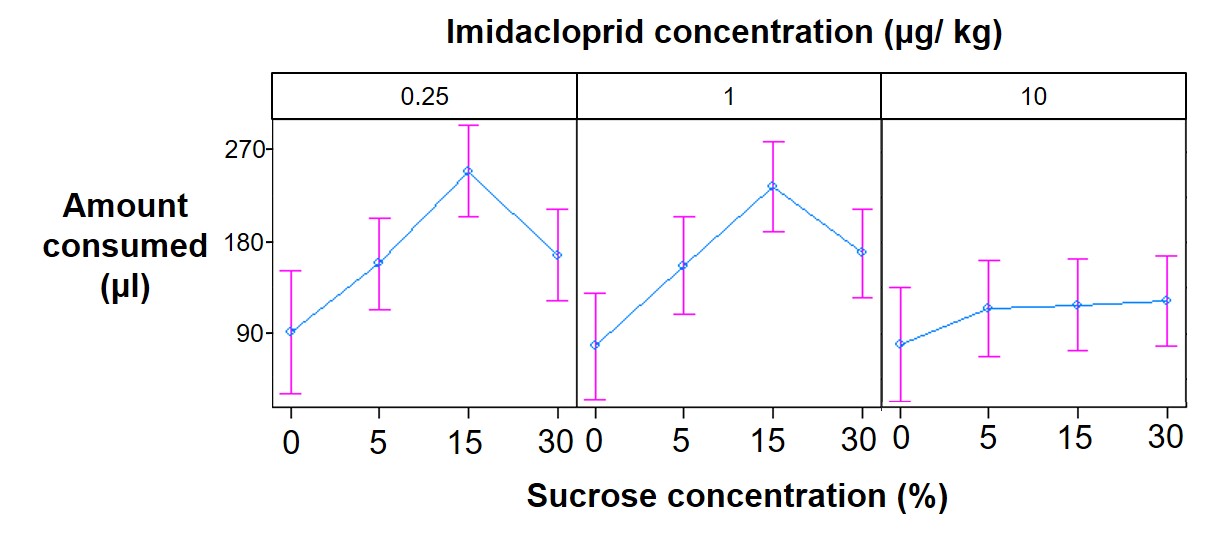


**Figure S3**: The mean ±SEM amount consumed in Exp. 1 after 24 hours for bees across all treatments.

**Experiment S1:** *Establishing that the concentration of Imidacloprid used in Exp. 1 has effects on preferences and feeding motivation*

*Methods*

In this experiment (carried out prior to Expt. 1), we assessed the effects of imidacloprid on feeding motivation and preference across a series of time points. Colonies (N =4, equally represented across treatments) were maintained and individuals were collected as described in the main text. Each subject was placed in a preference tube (as in Expt. 1) and two hours later offered two feeding tubes (plastic tubes sized ID × L: 3.56 × 65mm; note these feeding tubes are smaller than what we used in Expt. 1). Feeding tubes were spaced 5mm apart and each filled with 500 µl of solution.

To determine whether imidacloprid concentration affected feeding motivation we carried out “no choice” treatments, where bees were offered two feeding tubes of the same solution: 30% (w/w) sucrose containing 0.25, 1, 5 or 10 µg/ kg (= 0.28, 1.12, 5.63 or 11.25 PPB). All treatments and sample sizes are summarised in Table S4. To determine whether bees preferred solutions containing neonicotinoid (and if this depended on imidacloprid concentration), we carried out “choice” treatments, where bees were offered one feeding tube with neonicotinoid-containing solution, and one feeding tube containing a control solution (the same except for no neonicotinoid present). We measured how much of each solution bees consumed (the distance the meniscus migrated in each preference tubes from its marked initial point) at the following time intervals: 15, 30, 60, 90 minutes, and 3, 4, 5, 7, and 24 hrs (1mm = 9.954 µl).

**Table S4**: Summary of treatments and final sample sizes used in Expt. S1. In all cases the sucrose used (“suc”) was 30% sucrose.


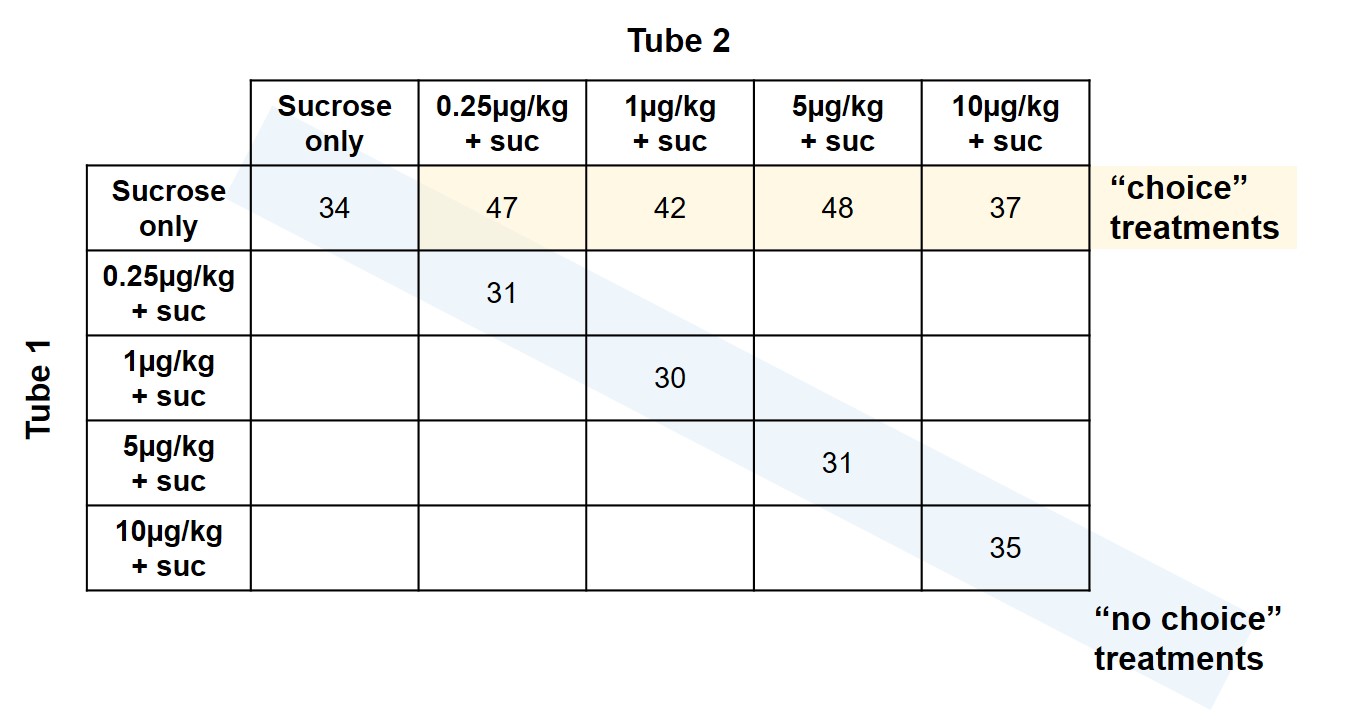


*Data Analyses*

To determine whether bees preferred to consume solutions that contained imidacloprid, and whether imidacloprid affected motivation to feed, we separately analysed the data of bees in “choice” and “no choice” treatments: the total consumption of solutions by bees in “no choice” treatments was used to determine feeding motivation while the relative consumption of the two solutions by bees in “choice” treatments was used to determine preference.

To determine whether the total consumption of solution was affected by imidacloprid concentration, we summed the amount consumed from both tubes in “no choice” treatments, and used non-cumulative values of sucrose consumption (i.e. how much sucrose had been consumed since the last measurement) to determine whether treatments varied in the amount they consumed across time. We ran LMMs (‘Model 5’) with the reponse variable “amount consumed” and the explanatory varibles “treatment” (control, 0.25, 1, 5 and 10µg/ kg), “time” (continuous) and the random factor “bee” nested in the random factor “colony”. We limited this analysis to the data from five time points: 90, 180, 240, 300, and 420 minutes. We did this because at time points previous to this, there were so many zeros (cases where the bees had not yet drunk anything), that the residuals from models were greatly skewed, and could not be transformed to be used in a parametric analysis. Similarly, consumption data at 24 hours was much greater than the rest of these values, and so we analysed this data separately, carrying out a single LMM (‘Model 6’) addressing the total amount consumed and the same explanatory variables described above (except for “time” and “bee”).

To determine whether bees preferred neonicotinoid-containing solutions over control solutions, and whether this differed by imidacloprid concentration and over time, we analysed the data from “choice” treatments and carried out a LMM (‘Model 7’) using the same variables and data selection as Model 5 (for the same reasons as described for Expt. 1, see main text), except that we also included the variable “solution type” (neonicotinoid or control). We analysed 24 hour data separately (‘Model 8’), using a LMM of the total amount consumed at 24 hours as the response variable and the same variables as Model 7 except for the variable “time”.

*Results*

In the no choice treatments, all bees consumed relatively less solution over time, but this effect was greater the higher the concentration of neonicotinoid in the solution (Model 5: treatment × time: *F_4, 409_* = 4.03, *p* < 0.005; time: *F_1, 409_* = 21.81, *p* < 0.0001; treatment: *F_4, 152_* = 0.29, *p* =0.89; Fig. S4). These differences first emerged at 3 hours, and were most apparent at 24 hours (Model 2: *F_4, 149_* = 30.73, *p* < 0.0001; post-hoc differences between groups shown on Fig. S4). In 19% of cases, bees consumed the total amount of solution available at 24 hours.

In the choice treatments, bees did not consume more of either the neonicotinoid-containing solution or the control solution (Model 7: *F_1, 1084_* = 0.99, *p* = 0.32) (addressing the amount consumed up to 7 hours). Bees also consumed relatively less solution over time (*F_1, 1084_* = 269.30, *p* < 0.0001), but the amount consumed was not affected by the imidacloprid concentration of the imidacloprid-containing solution (*F_3, 166_* = 1.85, *p* = 0.14). At 24 hours, bees also did not show a preference for one solution type over the other (Model 8: *F_1, 169_* = 0.50, *p* = 0.48), but bees tested in the highest-dose treatment consumed less overall than the other three treatments (*F_3, 162_* = 12.25, *p* < 0.0001; differences between groups confirmed with a Tukey post-hoc test). However, 28% of bees in the choice treatments consumed the total amount of each solution at 24 hours. To solve this issue, we used larger feeding tubes in Experiment 1 presented in the main text.


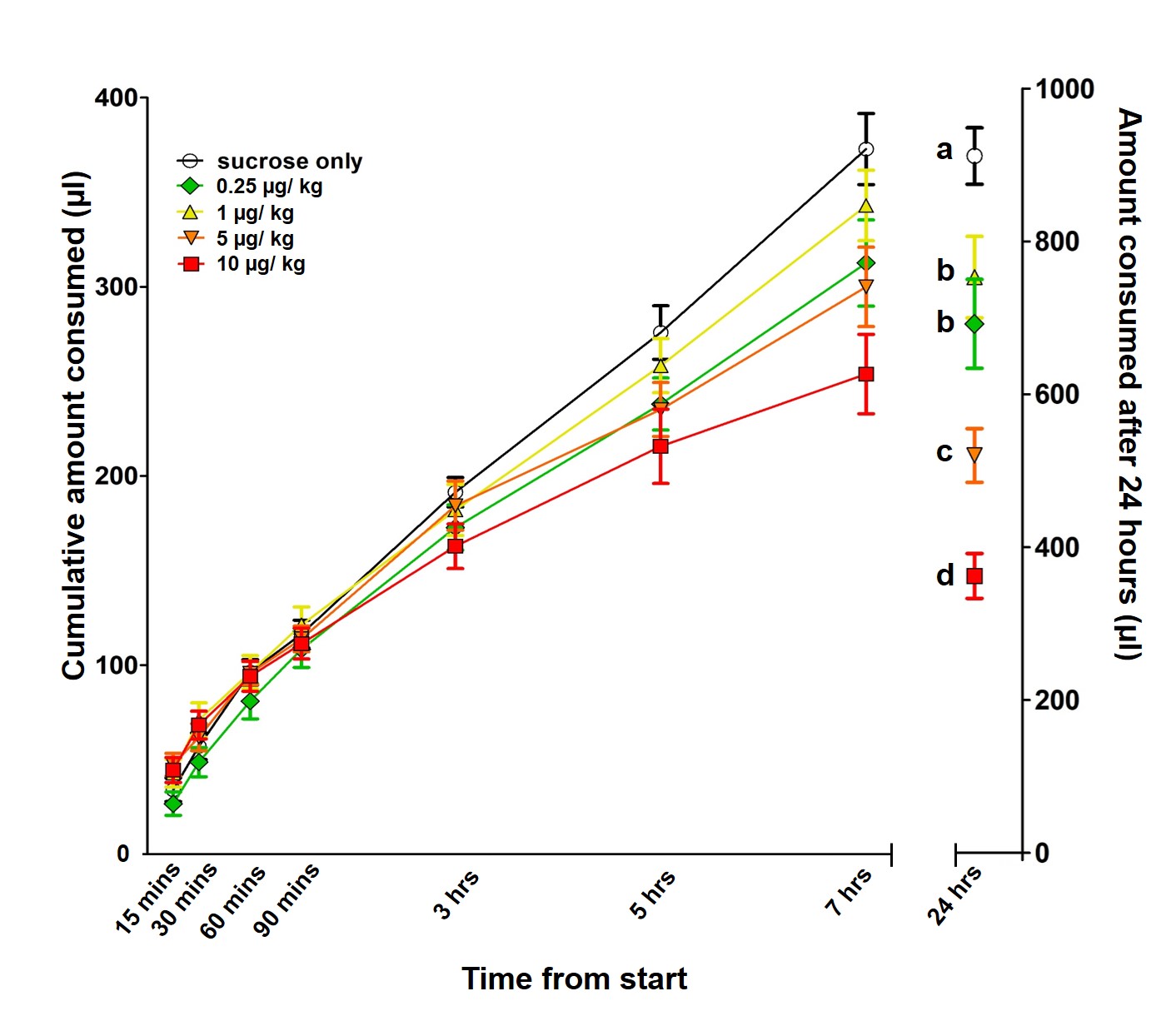


**Figure S4:** The mean (±SEM) amount of solution consumed over time in each of the five “no choice” treatments in Exp. S1where the two feeding tubes contained the same solution.

**Experiment S2:** *Establishing imidacloprid effects on locomotor* *activity*

*Methods*

To determine the effects of acute imidacloprid exposure on bumblebees’ short-term locomotor activity, we placed bees in transparent plastic square-ended tubes with ventilation holes (w×h×l = 2.5×2.5×13) sealed on either end, and let them sit for two hours before feeding each bee a single dose of either 0.2, 0.4, 1.1 or 2.2ng imidacloprid in 20 µl of a 30% (w/w) sucrose solution, or a control containing 10 or 100 µl acetone but no imidacloprid (we then pooled these control groups after finding no difference between them). 15 minutes after feeding we filmed bees in tubes for two hours. Tubes were filmed from above (using HD Sony Camcorders (30fps) mounted on tripods), with the centre of the tube marked with a black line. We coded each time the bee crossed the line during the two hour trial period as a measure of locomotor activity. Five bees did not move at all (control n=1; 0.2ng n=2; 1.1ng n=2), and were excluded from analyses, resulting in final sample sizes of: 0.2ng = 13; 0.4ng = 17; 1.1ng = 13; 2.2ng = 15; control = 23; these bees were taken from 7 colonies represented across all treatments.

*Data Analyses*

To determine how treatments differed in locomotor activity over time, we carried out a GLMM (‘Model 9’) with a Poisson distribution, with the response variable “number of crosses in a 15-minute time block”, and the explanatory variables “treatment” (control, 0.2, 0.4, 1.1 or 2.2ng) and “time” (continuous, 15-minute blocks from 15 to 120), and the random factor “bee” nested in the random factor “colony”.

*Results*

Overall, imidacloprid made bees initially less active in some treatments (0.4ng, 2.2ng), but then more active after a longer period of time had elapsed in all treatments (Model 9: treatment × time: *χ*^2^_4_ = 671.36; *p* < 0.0001; time: *χ*^2^_5_ = 11795; *p* < 0.0001; treatment: *χ*^2^_8_ = 677.76; *p* < 0.0001) (Fig. S5).


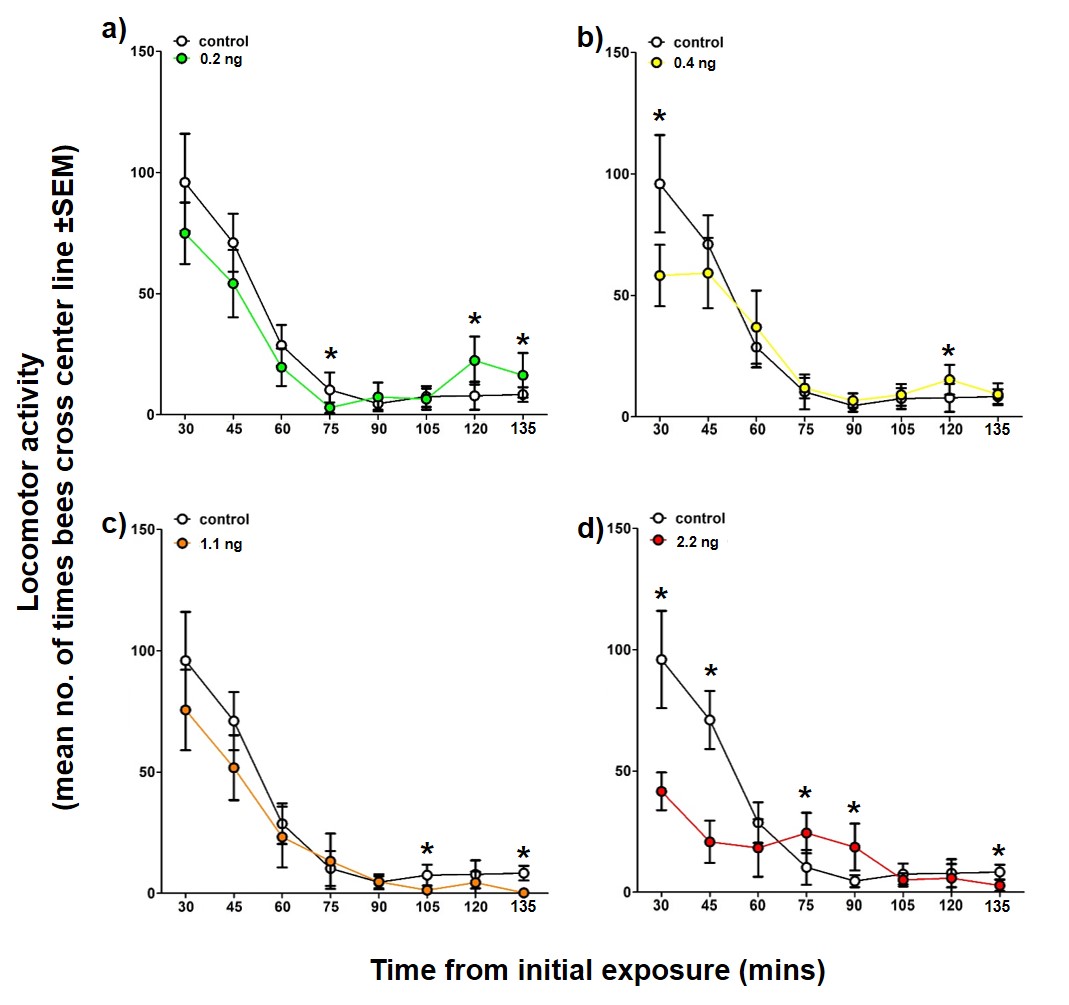


**Figure S5**: The locomotor activity of bees in Exp. S2 (measured as the mean ±SEM number of times bees cross a centre line), over the course of two hours for the four different treatment doses. We began filming at 15 minutes after initial exposure, thus the 30 minute data displayed here are activity from 15-30 mins post-exposure. Asterisks indicate significant differences between experimental and control treatments as revealed by a post-hoc test (control group shown is the same data for all treatments).

**Experiment S3:** *Testing for preferences in the dark, with the solutions more spatially separated*

*Methods*

After not finding preferences for imidacloprid in either Expt. S1 or Expt. 1, which differed to previous findings (Kessler et al., 2015), we aimed to determine why this might be. We identified three main factors that potentially differed between the previous experiment and our own: 1) the distance between the solutions being presented to bees; 2) the light conditions under which bees were tested; 3) the species being tested (*B. terrestris* compared to *B. impatiens*). We hypothesised that if bees cannot taste imidacloprid at the concentrations we used (as suggested by the electrophysiological data in Kessler et al., 2015), then any preferences would likely be due to post-ingestive effects. Post-ingestive effects may be more clearly detected if the two solutions were spaced further apart and bees were tested in the dark, since this would mean a greater amount of time between sampling the first and second solution (see Discussion). Thus, we repeated our preference trials, more closely replicating the conditions of Kessler et al. (2015): we collected bees as they exited their colony to forage, cold-anaesthetized them and placed them into preference containers (l×w×h= 22 x 15 x 5.5 cm) under dark conditions. These preference containers already contained feeding tubes (of the same size as in Expt 1), inserted into either end of the container i.e. spatially separated by 22cm. We then left bees in the dark until we measured the amount of solution consumed from each of the feeding tubes after 24 hours (rather than multiple measures across time as in Expt. S1 and 1). We carried out this experiment using the dose that was closest to what was found to have the largest effect in Kessler et al. (2015): 0.26 PPB IMD in 15% (w/w) sucrose (they used 1 nM IMD in 0.5 M sucrose (0.256 PPB and ~17% sucrose) vs. the control solution (no IMD) of the same sucrose concentration. We tested 48 bees from two colonies. We also carried out 6 controls for evaporation (i.e. 12 feeding tubes total), carried out in the same way as experimental treatments but without bees present. We took an average of the evaporation for each solution type, and subtracted it from the raw data for each bee. Bees that consumed less than 10ul in total (after controlling for evaporation) were removed (n=1).

*Data Analyses*

To determine whether bees differed in how much pesticide-containing solution they consumed relative to the control solution, we carried out a LMM (‘Model 10’) with the response variable “amount consumed” and the explanatory variable “solution type” with the random factor “bee” nested in the random factor “colony”.

*Results*

Bees did not show a preference for one solution over the other, consuming the same amount of both solutions (Model 10: *F*_1, 46_ = 0.75; *p* = 0.39; Fig. S6).


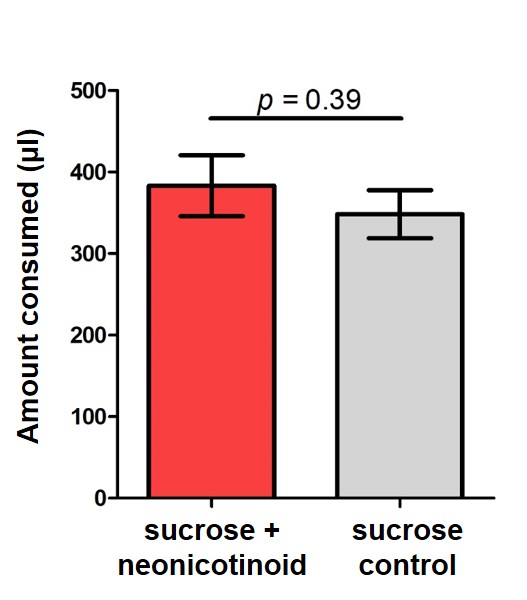


**Figure S6**: The mean ±SEM amount consumed of the two possible solutions in Exp. S3 when bees were tested in the dark after 24 hours.

**Experiment S4**: *Demonstration of learning in the protocol used for Expt. 2*

*Methods*

To ensure that bees could learn in the protocol we used in Expt. 2, we carried out a ‘proof of concept’ experiment involving identical methods, with the exception of the composition of the two solutions offered (neonicotinoid-free 10% sucrose or 50% sucrose). We presented half the bees (N = 10) with the 50% solution first, and half (N = 10) with the 10% solution first. One bee died and one bee did not consume the sucrose presented to it on the first trial, resulting in n = 18 bees. All of these bees consumed the solution presented to it on every trial.

One hour after the final presentation, we gave bees a test (probe) trial carried out in the same way as Expt. 2 (Fig. S7). We recorded the total time the bee spent on each stimulus in the five-minute observation period. This was defined as the bee having, at a minimum, their head and front two legs on the stimulus; we did not include cases where the bee was oriented such that their head was pointed away from the stimulus and only their back legs or abdomen was on the stimulus. Also, if a bee went to a stimulus but then lay still, unmoving, we ceased counting its time on the stimulus after it stopped moving. We also initially recorded when the bee’s proboscis extended to the droplet, but we did not use this measure in analysis since it could not be seen reliably from above.


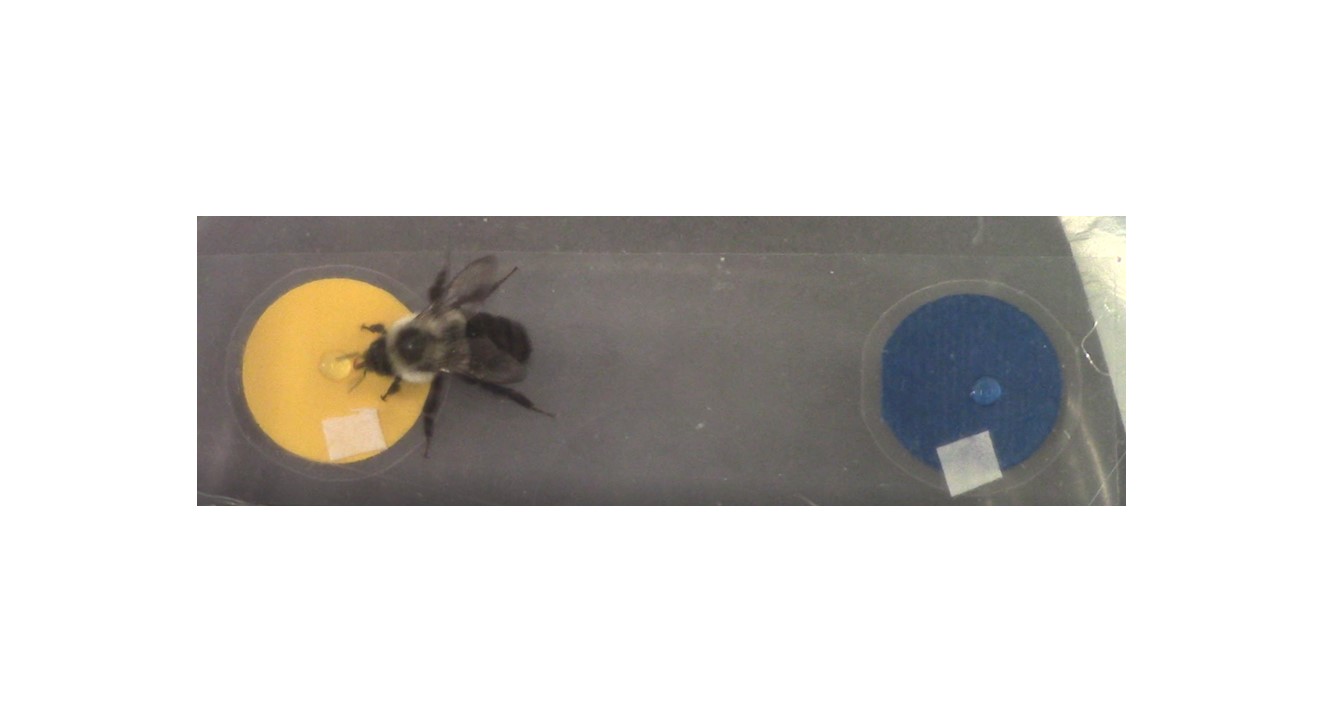


**Fig. S7:** Screenshot of a video of a bees’ test (probe) trial in Exp. S4 where the bee was presented with both stimuli containing water droplets. Each stimulus contained three elements: colour (blue or yellow), scent (geraniol or eugenol, on a piece of filter paper) and location (left or right).

*Data Analyses*

To determine whether bees learned to associate a stimulus with a higher concentration sucrose reward (50% over 10%) that it was paired with, we addressed the total amount of time (in a 5-minute observation period) that bees spent exploring a given stimulus. We carried out two LMMs (one for the data after 1 minute, one for the data after 5 minutes; ‘Model 11’) with the response variable of the total time on the stimulus (seconds) and the explanatory factor “reward quality” (50% (i.e. CS+) or 10%). We also included the fixed factors “colour paired with CS+” (blue or yellow), “scent paired with CS+” (geraniol or eugenol), “location of CS+” (left or right), and the random factor “bee”.

*Results*

Bees learned which stimulus had previously been paired with the superior reward, spending more time on the cue that had previously been paired with the 50% solution than the one that had previously been paired with. This was the case both after 1 minute (*F*_1, 17_ = 14.10, *p* < 0.005) and after five minutes (*F*_1, 17_ = 13.65, *p* < 0.005). We also addressed bees’ ‘first choice’, measured as the first cue that bees approached and extended their proboscis to. However, this was not a useful measure of learning, since bees seemed to approach whichever cue they were closest to first: 10 bees approached the cue previously paired with 50% first, while 8 bees approached the other cue first. We thus did not use this measure in Expt. 2.

**References**

Douglas Bates, Martin Maechler, Ben Bolker, Steve Walker (2015). Fitting Linear Mixed-Effects Models Using lme4. *Journal of Statistical Software*, 67(1), 1-48. doi:10.18637/jss.v067.i01.

Fox, J. (2003). Effect Displays in R for Generalised Linear Models. *Journal of Statistical Software*, 8(15), 1-27. URL http://www.jstatsoft.org/v08/i15/.

Kessler, S. C., Tiedeken, E. J., Simcock, K. L., Derveau, S., Mitchell, J., Softley, S., … Wright, G. A. (2015). Bees prefer foods containing neonicotinoid pesticides. *Nature*, *521*(7550), 74–76. https://doi.org/10.1038/nature14414

R Core Team, (2017). R: A language and environment for statistical computing. R Foundation for Statistical Computing, Vienna, Austria. URL http://www.R-project.org/.

Pinheiro J, Bates D, DebRoy S, Sarkar D, R Core Team (2018). _nlme: Linear and Nonlinear Mixed Effects Models_. R package version 3.1-137, <URL: https://CRAN.R-project.org/package=nlme>.

Russell Lenth (2018). emmeans: Estimated Marginal Means, aka Least-Squares Means. R package version 1.2.2. https://CRAN.R-project.org/package=emmeans
